# Clinical Validation of Human Papilloma Virus Circulating Tumor DNA for Early Detection of Residual Disease After Chemoradiation in Cervical Cancer

**DOI:** 10.1200/JCO.23.00954

**Published:** 2023-11-16

**Authors:** Kathy Han, Jinfeng Zou, Zhen Zhao, Zeynep Baskurt, Yangqiao Zheng, Elizabeth Barnes, Jennifer Croke, Sarah E. Ferguson, Anthony Fyles, Lilian Gien, Adam Gladwish, Magali Lecavalier-Barsoum, Stephanie Lheureux, Jelena Lukovic, Helen Mackay, Eve-Lyne Marchand, Ur Metser, Michael Milosevic, Amandeep S. Taggar, Scott V. Bratman, Eric Leung

**Affiliations:** ^1^ Princess Margaret Cancer Centre, University Health Network, Toronto, Ontario, Canada; ^2^ Department of Radiation Oncology, University of Toronto, Toronto, Ontario, Canada; ^3^ Department of Biostatistics, University Health Network, Toronto, Ontario, Canada; ^4^ Odette Cancer Centre, Sunnybrook Health Sciences Centre, Toronto, Ontario, Canada; ^5^ Department of Obstetrics and Gynecology, University of Toronto, Toronto, Canada; ^6^ Jewish General Hospital, Montreal, Quebec, Canada; ^7^ Division of Medical Oncology, Department of Internal Medicine, University of Toronto, Toronto, Ontario, Canada; ^8^ Hopital Maisonneuve-Rosemont, Montreal, Quebec, Canada; ^9^ Joint Department of Medical Imaging, University Health Network, University of Toronto, Toronto, Ontario, Canada

## Abstract

**PURPOSE:**

Most cervical cancers are caused by human papilloma virus (HPV), and HPV circulating tumor DNA (ctDNA) may identify patients at highest risk of relapse. Our pilot study using digital polymerase chain reaction (dPCR) showed that detectable HPV ctDNA at the end of chemoradiation (CRT) is associated with inferior progression-free survival (PFS) and that a next-generation sequencing approach (HPV-seq) may outperform dPCR. We aimed to prospectively validate HPV ctDNA as a tool for early detection of residual disease.

**METHODS:**

This prospective, multicenter validation study accrued patients with stage IB-IVA cervical cancer treated with CRT between 2017 and 2022. Participants underwent phlebotomy at baseline, end of CRT, 4-6 weeks post-CRT, and 3 months post-CRT for HPV ctDNA levels. Plasma HPV genotype–specific DNA levels were quantified using both dPCR and HPV-seq. The primary end point was 2-year PFS.

**RESULTS:**

With a median follow-up of 2.2 (range, 0.5-5.5) years, there were 24 PFS events among the 70 patients with HPV+ cervical cancer. Patients with detectable HPV ctDNA on dPCR at the end of CRT, 4-6 weeks post-CRT, and 3 months post-CRT had significantly worse 2-year PFS compared with those with undetectable HPV ctDNA (77% *v* 51%, *P* = .03; 82% *v* 15%, *P* < .001; and 82% *v* 24%, *P* < .001, respectively); the median lead time to recurrence was 5.9 months. HPV-seq showed similar results as dPCR. On multivariable analyses, detectable HPV ctDNA on dPCR and HPV-seq remained independently associated with inferior PFS.

**CONCLUSION:**

Persistent HPV ctDNA after CRT is independently associated with inferior PFS. HPV ctDNA testing can identify, as early as at the end of CRT, patients at high risk of recurrence for future treatment intensification trials.

## Teaser

Persistent HPV ctDNA following chemoradiation is associated with inferior PFS in cervical cancer.

## INTRODUCTION

Despite chemoradiation (CRT), 30%-40% of patients with locally advanced cervical cancer relapse.^1^ Accurate prognostication allows robust stratification in clinical trials and personalized treatment to improve outcome. Clinical prognostic factors (such as stage and lymph node status) identified to date are poor predictors of relapse with a concordance index of approximately 0.62.^2^

**CONTEXT**

**Key Objective**
Does human papilloma virus circulating tumor DNA (HPV ctDNA) predict relapse in patients with locally advanced cervical cancer after definitive chemoradiation (CRT)?
**Knowledge Generated**
A sequencing-based assay (HPV-seq) enables highly sensitive detection and accurate HPV genotyping from baseline plasma cell-free DNA. Detectable HPV ctDNA at each of the following three timepoints is independently associated with inferior progression-free survival (PFS): end of CRT, 4-6 weeks post-CRT, and 3 months post-CRT. Despite higher sensitivity of HPV-seq, there is no statistically significant difference in the performance (concordance index) of HPV-seq versus HPV genotype–matched digital polymerase chain reaction in predicting PFS.
**Relevance *(G. Fleming)***
Useful salvage therapy is not currently available for the majority of relapsed cervical cancer patients. These results should be useful in selecting the highest risk patients for the development of treatments to prevent clinical recurrence.*
  *Relevance section written by *JCO* Associate Editor Gini Fleming, MD.

Most cervical cancers are caused by human papilloma virus (HPV), providing a convenient genetic marker of cancer-derived DNA that could be used to assess residual disease burden within plasma. Detection of HPV circulating tumor DNA (ctDNA) after CRT may identify patients at highest risk of relapse. Our previous pilot study using digital polymerase chain reaction (dPCR) showed that detectable HPV ctDNA at the end of CRT is associated with inferior progression-free survival (PFS)^3^ and that a next-generation sequencing approach (HPV-seq) using dual-strand full-length viral capture may outperform dPCR.^4^ While other studies have also shown an association between HPV ctDNA and outcome in cervical cancer, many are from samples of convenience from varying timepoints, retrospective nature, and/or smaller cohort size.^5-12^ We hypothesized that HPV ctDNA may identify patients with cervical cancer at increased risk of relapse after CRT and aimed to prospectively validate HPV ctDNA as a tool for early detection of molecular residual disease (MRD).^13^

## METHODS

### Patients and Study Design

This prospective, multicenter study recruited patients to validate HPV ctDNA as a biomarker and compare with positron emission tomography (PET) imaging across four Canadian centers between October 2017 and June 2022. It was approved by the local Institutional Review Boards. Female patients older than 18 years with histologically confirmed 2009 International Federation of Gynecology and Obstetrics (FIGO) stage IB2-IVA cervical squamous cell carcinoma, adenocarcinoma, or adenosquamous carcinoma planned for definitive CRT were eligible (see the Data Supplement, online only, for more details). Stage was reclassified using the revised 2018 FIGO staging system. All participants provided written informed consent before enrollment and did not receive compensation for the study. No commercial support was provided. The study was registered with ClinicalTrials.gov (identifiers: NCT03853915 and NCT03702309).

Patients were examined every 3-4 months during the first 2 years after CRT and every 6 months during years 3-5 for disease status. Imaging was performed 3-4 months post-CRT (Data Supplement). Relapses were documented by clinical examination and imaging and wherever feasible also by histologic confirmation. The follow-up data cutoff was March 2, 2023.

### Blood Collection and ctDNA Analysis

As part of the study, patients underwent phlebotomy at baseline (before CRT), end of CRT, 4-6 weeks post-CRT, and 3 months post-CRT for HPV ctDNA levels. At each timepoint, 30 mL of peripheral blood was collected in Streck Cell-Free DNA BCT tubes, shipped to and processed for plasma by a central laboratory (median, 1 day; IQR, 0-2 days), and stored at −80°C. Cell-free DNA extraction and quality assessment were performed as previously described.^3^ HPV genotyping was performed on the baseline plasma sample using HPV-seq, which targets 38 distinct HPV types; HPV genotype–specific DNA levels in plasma were quantified using both dPCR and HPV-seq as previously described.^3,4^ For copy number quantification using HPV-seq, a normalization factor of 0.6 was applied to account for inflation of HPV-mapped reads relative to human-mapped reads because of incorporation of dual-stranded HPV hybrid capture baits.^4^ Study personnel who quantified HPV ctDNA were blinded to clinical characteristics and outcomes.

### Fluorodeoxyglucose PET Imaging

As part of the study, patients enrolled after April 2019 (after external funding was secured) also underwent fluorodeoxyglucose (FDG)-PET/computed tomography imaging 3 months after CRT. Tumor response on PET imaging was defined according to previous studies.^3,14^

### Statistical Analysis

The primary end point was PFS, defined as the time from histologic diagnosis of cervical cancer to the time of relapse or death. The primary objective was to detect a difference in 2-year PFS between those with detectable versus undetectable HPV ctDNA after CRT. The sample size was calculated using a 2-year PFS rate of 70% from our large institutional series^15^ and assuming that 30% of the patients would have detectable HPV ctDNA post-CRT on the basis of data from our pilot study. With a one-sided significance level at 0.05, accruing 84 patients over 2 years with an additional follow-up of 2 years (accounting for 15% dropout and/or unanalyzable HPV-negative/undetectable cases) would provide an 86% power to detect an effect size (hazard ratio [HR]) of 3 and a 96% power to detect a stronger HR of 4. Because of the uncertainty regarding accrual rate and funding, the plan was originally to include 20 patients from the pilot study. However, the relatively large number of centers that participated in the study subsequently warranted dropping the 20 patients from the pilot study. Thus, the present study consists of 84 patients newly accrued into this validation study.

PFS was estimated using the Kaplan-Meier method and compared using the log-rank test. Univariable analysis for PFS was performed using the Cox proportional hazard model, considering the following variables: age, stage, histology, and detectable HPV ctDNA at end of CRT, 4-6 weeks post-CRT, or 3 months post-CRT. Variables with *P* < .05 were included in multivariable Cox regression analyses to determine independent prognostic factors associated with PFS. Sensitivity was defined as the percentage of patients who relapsed in the follow-up period who were ctDNA-positive at the specified timepoint(s).^16^ Specificity was defined as the percentage of patients who did not relapse in the follow-up period and who were ctDNA-negative at the specified timepoint(s).^16^

For the secondary objective of determining the accuracy of HPV ctDNA versus PET imaging, the C-index was estimated using the *coxph* function in survival R package and compared using a z test where the variance-covariance matrix for the C-index values was obtained from vcov and concordance functions. All *P* values were two-sided with a significance level of <.05 for the primary log-rank and Cox regression analyses. Bonferroni correction was applied to account for multiple comparisons of C-indices. R version 4.2.2 was used for all analyses.

## RESULTS

### Prospective Validation Cohort and Outcomes

**FIG 1.**
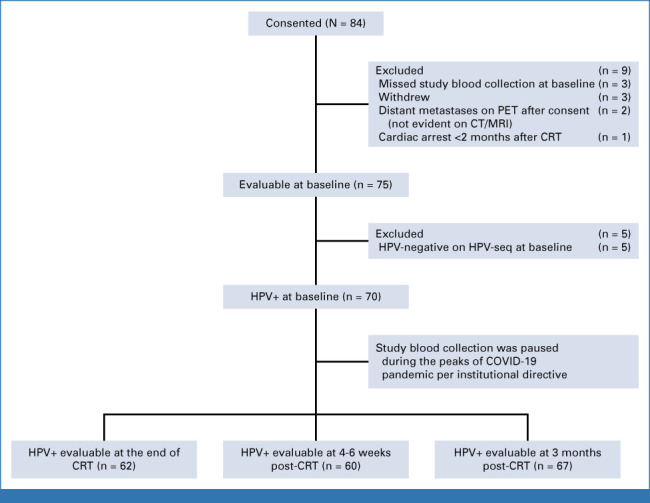
Flow diagram. CRT, chemoradiation; CT, computed tomography; HPV, human papilloma virus; MRI, magnetic resonance imaging; PET, positron emission tomography.

Between October 2017 and June 2022, 84 patients were enrolled and 75 were evaluable (Fig 1). Study blood collection was paused during the peaks of the COVID-19 pandemic as per the institutional directive to preserve clinical capacity. Patient, tumor, and treatment characteristics are summarized in Table 1. All patients received external beam radiotherapy with brachytherapy boost, and 97% received concurrent cisplatin. With a median follow-up of 2.2 years (range, 0.5-5.5 years), the 2-year PFS was 66% (95% CI, 56 to 79). There were 24 PFS events. Most relapses (15 of 24, 63%) included distant metastasis: 11 distant, one distant and para-aortic nodal, three distant and local, five paraaortic nodal, one pelvic/paraaortic nodal and local, two pelvic nodal, and one local.

**TABLE 1.** Patient, Tumor, and Treatment Characteristics

Characteristic	n = 70
Age, years, mean ± standard deviation	54 ± 15
2018 FIGO stage, No. (%)	
IB	6 (9)
IIA	8 (11)
IIB	21 (30)
IIIA	1 (1)
IIIB	2 (3)
IIIC1	17 (24)
IIIC2	7 (10)
IVA	8 (11)
Histologic type, No. (%)	
Squamous cell carcinoma	59 (84)
Adenocarcinoma or adenosquamous carcinoma	11 (16)
Tumor size at diagnosis, cm, median (range)	4.6 (2.1-9.1)
Cisplatin chemotherapy receipt, No. (%)	68 (97)
Brachytherapy receipt (28 Gy/4F or 24 Gy/3F), No. (%)	70 (100)
Brachytherapy technique	
Magnetic imaging resonance–guided	67 (96)
CT-guided	3 (3)
CTV_HR_ D_90%_ (Gy_10_), mean ± standard deviation	91.3 ± 4.5

Abbreviations: CT, computed tomography; CTV_HR_ D_90%_, minimum combined external beam radiotherapy and brachytherapy equivalent dose in 2 Gy fractions to 90% of the high-risk clinical target volume; FIGO, International Federation of Gynecology and Obstetrics.

### HPV Genotyping From Baseline Plasma

Of the 75 evaluable patients, HPV-seq detected HPV ctDNA and provided HPV genotype information directly from the baseline plasma sample of 70 patients. The majority (38 of 70, 54%) harbored HPV-16, and 46% harbored other HPV types: 15 HPV-18; five HPV-59; two each HPV-31, HPV-33, HPV-45, and HPV-52; and one each HPV-39, HPV-53, HPV-58, and HPV-82. Nine of the 70 patients with detectable HPV ctDNA at baseline also had clinical HPV testing on cervical tumor biopsy: eight had concordant genotypes between HPV-seq and tumor tissue analyses (one HPV-16, three HPV-18, one HPV-33, one HPV-52 and one HPV-53, and one HPV-59) and one had no HPV DNA detected from tumor biopsy but HPV-18 ctDNA in plasma at all timepoints (P11: 12 copies/mL at baseline, up to 158 copies/mL at 3 months). Of the five patients with undetectable HPV ctDNA in the baseline plasma sample, clinical HPV testing on three patients' diagnostic (baseline) cervical biopsy was performed. All three patients' biopsy specimens were HPV-negative as well.

Of the 70 patients genotyped by HPV-seq, 65 (93%) were validated by dPCR, whereas five had undetectable HPV ctDNA at baseline on dPCR. These five patients had low levels (range, 0.29-17.3 copies/mL) of HPV ctDNA by HPV-seq, suggesting that these may represent false-negative dPCR results with levels below the detection threshold.

### HPV ctDNA Quantification and MRD Detection

**FIG 2.**
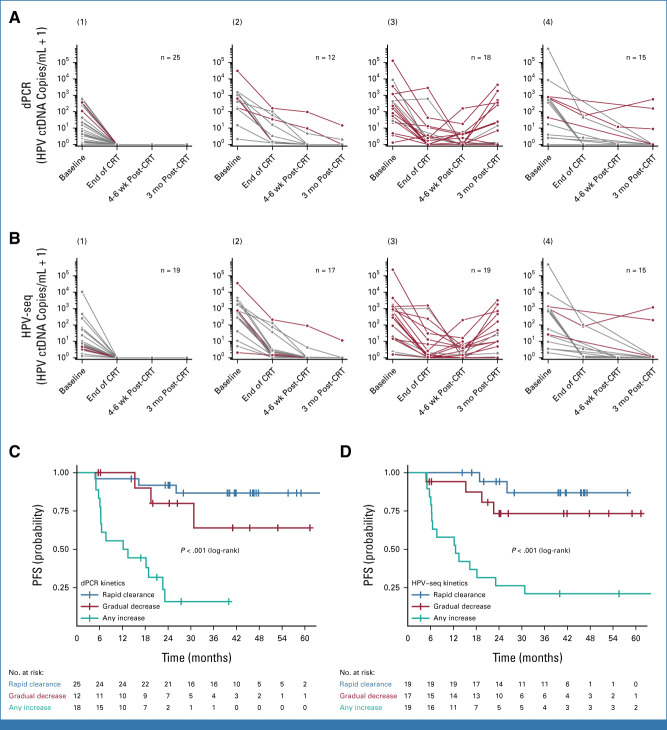
HPV ctDNA burden over time on the basis of (A) dPCR and (B) HPV-seq. Patients who are disease-free are depicted in gray; those who relapsed are depicted in red. The change in HPV ctDNA burden was determined using adjacent timepoints. Patients had (1) rapid clearance of HPV ctDNA at all three nonbaseline timepoints (end of CRT, 4-6 weeks post-CRT, and 3 months post-CRT); (2) a gradual decrease in HPV ctDNA; (3) an increase in HPV ctDNA level relative to an earlier adjacent timepoint; or (4) missing adjacent timepoints. PFS by HPV ctDNA kinetic patterns (1)-(3), on the basis of (C) dPCR and (D) HPV-seq. CRT, chemoradiation; dPCR, digital polymerase chain reaction; HPV ctDNA, human papilloma virus circulating tumor DNA; PFS, progression-free survival.

The HPV ctDNA burden/kinetic pattern is shown in Figure 2 and the Data Supplement. Any increase in HPV ctDNA was associated with worse PFS. HPV ctDNA levels from HPV-seq were highly correlated with dPCR results (*R*^2^ = 0.99; 95% CI, 0.90 to 1.00; *P* < 10^−66^; Data Supplement). The lowest detectable level was 0.18 copy/mL on dPCR and 0.023 copy/mL on HPV-seq. Baseline HPV ctDNA levels were higher for stage III/IVA compared with those for stage IB/II disease (Data Supplement). The Data Supplement shows serial HPV ctDNA detectability and clinical outcome. Median lead time from the first positive HPV ctDNA test to identification of relapse by clinical examination/radiologic imaging was 5.9 months (range, 0-28 months) for both dPCR and HPV-seq (Data Supplement).

**FIG 3.**
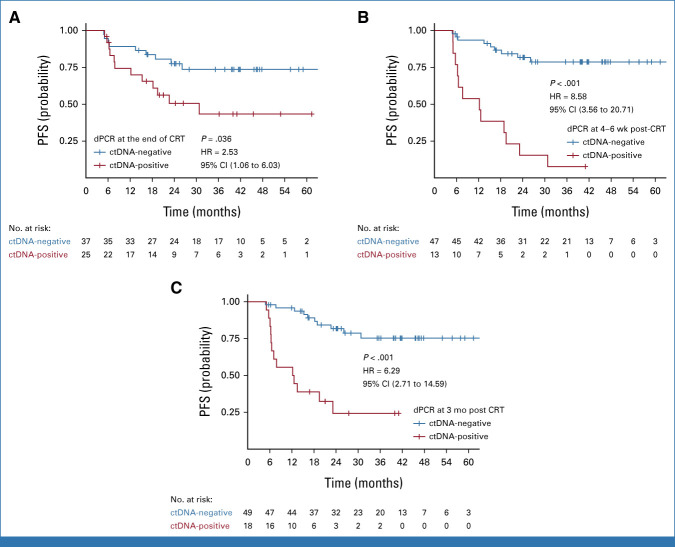
PFS by HPV DNA copy number in plasma on dPCR at (A) end of CRT, (B) 4-6 weeks post-CRT, and (C) 3 months post-CRT. CRT, chemoradiation; ctDNA, circulating tumor DNA; dPCR, digital polymerase chain reaction; HPV, human papilloma virus; HR, hazard ratio; PFS, progression-free survival.

**FIG 4.**
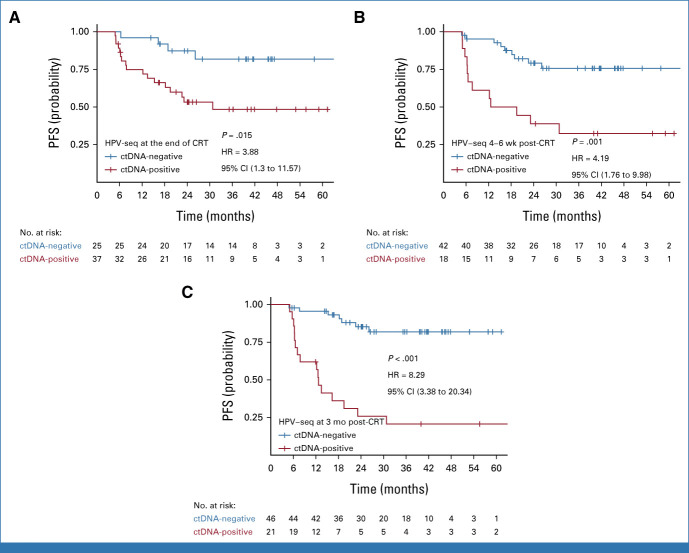
PFS by HPV DNA copy number in plasma on HPV-seq at (A) end of CRT, (B) 4-6 weeks post-CRT, and (C) 3 months post-CRT. CRT, chemoradiation; ctDNA, circulating tumor DNA; HPV, human papilloma virus; HR, hazard ratio; PFS, progression-free survival.

Patients with detectable HPV ctDNA on dPCR at the end of CRT, 4-6 weeks post-CRT, and 3 months post-CRT had significantly worse 2-year PFS compared with those with undetectable HPV ctDNA (Fig 3; 77% *v* 51%, *P* = .03; 82% *v* 15%, *P* < .001; and 82% *v* 24%, *P* < .001, respectively). HPV-seq showed similar results (Fig 4; 87% *v* 53%, *P* = .009; 79% *v* 39%, *P* < .001; and 85% *v* 26%, *P* < .001, respectively). Results of univariable Cox regression analysis are shown in Table 2. On multivariable analyses, detectable HPV ctDNA on dPCR and HPV-seq remained independently associated with inferior PFS (Table 2).

**TABLE 2.** Univariable and Multivariable Regression for Progression-Free Survival

Variable	Univariable Regression					
	dPCR			HPV-Seq		
	HR (95% CI)	*P*	C-Index	HR (95% CI)	*P*	C-Index
Age	0.99 (0.96 to 1.02)	.400	0.56	Same		
Histology						
Squamous cell carcinoma	Reference			Same		
Nonsquamous cell carcinoma	1.18 (0.44 to 3.13)	.740	0.49			
2018 FIGO stage						
IB/II	Reference			Same		
III/IVA	2.90 (1.20 to 7.01)	.020	0.63			
Detectable HPV ctDNA at the end of CRT	2.53 (1.06 to 6.03)	.040	0.61	3.88 (1.30 to 11.57)	.010	0.65
Detectable HPV ctDNA at 4-6 weeks post-CRT	8.58 (3.56 to 20.71)	<.001	0.72	4.19 (1.76 to 9.98)	.001	0.68
Detectable HPV ctDNA at 3 months post-CRT	6.29 (2.71 to 14.59)	<.001	0.72	8.29 (3.38 to 20.34)	<.001	0.75

Model	Multivariable Regression					
	dPCR			HPV-Seq		
	HR (95% CI)	*P*	C-Index	HR (95% CI)	*P*	C-Index
2018 FIGO stage III/IVA *v* IB/II	3.12 (1.21 to 8.09)	.020	0.70	3.43 (1.32 to 8.89)	.010	0.73
Detectable HPV ctDNA at the end of CRT	2.44 (1.02 to 5.82)	.045		4.13 (1.38 to 12.34)	.010	
2018 FIGO stage III/IVA *v* IB/II	1.91 (0.69 to 5.27)	.210	0.80	3.06 (1.18 to 7.93)	.020	0.76
Detectable HPV ctDNA at 4-6 weeks post-CRT	6.79 (2.66 to 17.34)	<.001		3.86 (1.61 to 9.22)	.002	
2018 FIGO stage III/IVA *v* IB/II	1.84 (0.73 to 4.59)	.190	0.75	2.16 (0.88 to 5.33)	.090	0.78
Detectable HPV ctDNA at 3 months post-CRT	5.50 (2.32 to 13.04)	<.001		7.78 (3.15 to 19.24)	<.001	

Abbreviations: CRT, chemoradiation; ctDNA, circulating tumor DNA; dPCR, digital polymerase chain reaction; FIGO, International Federation of Gynecology and Obstetrics; HPV, human papilloma virus; HR, hazard ratio.

When the analysis was repeated excluding the five patients who had undetectable dPCR at baseline (but detectable on HPV-seq), similar results were obtained (data not shown).

### Accuracy of MRD Detection Using HPV ctDNA

The sensitivity, specificity, and C-index of HPV-seq and dPCR for predicting PFS are shown in the Data Supplement. There were no significant differences in the performance (C-indices) of HPV-seq versus dPCR in predicting PFS (*P* = .42 for the end of treatment, *P* = .22 for 4-6 weeks, and *P* = .34 for 3 months). There was also no significant difference in the performance at the end-of-treatment versus 4-6 week timepoint for HPV-seq (*P* = .91). C-index for 4- 6 week dPCR was higher than that for the end-of-treatment dPCR (0.71 *v* 0.60, *P* = .04) but not statistically significant after accounting for multiple comparisons.

There were 17 PFS events among the subset of 44 patients who underwent both 3-month FDG-PET imaging and blood collection (Data Supplement). C-indices of FDG-PET imaging, dPCR HPV ctDNA, and HPV-seq HPV ctDNA at 3 months for predicting relapse at 2 years were 0.75, 0.65, and 0.74, respectively (*P* = .14 for PET *v* dPCR; *P* = .85 for PET *v* HPV-seq). Of note, while four patients' PET scans were reported as showing suspicious uptake in the cervix or lower vagina, they ultimately relapsed distantly with no evidence of disease in the cervix or vagina (ie, false-positive PET scan scored as predicting relapse). Representative examples are shown in the Data Supplement.

Given the fact that studies in other disease sites show that ctDNA sensitivity for predicting recurrence can be augmented via a longitudinal surveillance approach (ie, blood draws at multiple timepoints during follow-up with ctDNA status determined by whether any blood draw is positive),^16^ we evaluated the performance of HPV ctDNA detectability at any timepoint (end of treatment, 4-6 weeks post-treatment, and/or 3 months post-treatment). This longitudinal surveillance approach resulted in higher sensitivity but lower specificity compared with the single timepoint landmark approach presented above (Data Supplement).

## DISCUSSION

To our knowledge, this is the first study that prospectively validates HPV ctDNA as a tool for early detection of residual disease after CRT in patients with locally advanced cervical cancer. It uses the same methodology and eligibility criteria as our pilot study.^3^ Persistent HPV ctDNA after CRT was independently associated with inferior PFS in this prospective validation study at all timepoints, thus confirming our previous results. There was no statistically significant difference between the performance of HPV-seq and dPCR in MRD detection nor between the timepoints that were evaluated, highlighting the benefit of early identification of patients with MRD at risk of relapse.

In our initial study describing analytical and clinical performance attributes of HPV-seq, we had found 100% accuracy of HPV genotyping among 10 patients with matched plasma HPV-seq and clinical tissue testing.^4^ In the present study, HPV-seq again provided high accuracy for genotyping. Moreover, HPV-seq was able to detect and genotype HPV ctDNA from baseline plasma in a patient with a negative result on clinical tissue testing. These results confirm our previous findings that HPV-seq using a broad genotyping panel could replace tumor tissue analysis for accurate HPV genotyping.^17^ Sastre-Garau et al also reported high sensitivity (95%) using their sequencing-based CaptHPV approach to detect HPV ctDNA in blood samples compared with matched tumor tissue at baseline.^5^

The finding that HPV-seq and dPCR had similar overall accuracy in the MRD setting was unexpected on the basis of our previous findings that HPV-seq has superior analytical sensitivity.^4^ In the present study, we again noted that HPV-seq detected lower levels of HPV ctDNA (lowest level detected 0.023 copies/mL; 100% sensitivity at baseline) compared with dPCR (lowest level detected 0.18 copies/mL; 93% sensitivity at baseline). Nonetheless, modest numerical improvements in sensitivity for MRD at the various post-treatment timepoints appeared to be largely offset by reduced specificity of HPV-seq versus dPCR. Studies in HPV-positive oropharynx cancer using dPCR have demonstrated incomplete HPV ctDNA clearance at timepoints before 3 months post-CRT,^18,19^ raising the possibility that a more sensitive assay such as HPV-seq could be more susceptible to remnants of treated disease before it is completely cleared. Although we counted any detectable HPV ctDNA as a positive result, future studies could seek to optimize cutoff values that maximize either sensitivity or specificity depending on the clinical use case.

Overall, we found no statistically significant differences in relapse prediction between the different timepoints or compared with 3-month PET imaging. Early post-CRT timepoints, that is, within the standard adjuvant treatment window while residual disease burdens are lower, are likely of greater clinical utility. As such, this study included a 4- to 6-week post-treatment timepoint in addition to the end-of-treatment and 3-month post-treatment timepoints from our pilot study. Although there is currently no recommended adjuvant regimen after definitive CRT for cervical cancer, there remains a strong clinical unmet need because of poor outcomes among patients who recur.^20^ With the recent failure of large studies that included patients on the basis of the pretreatment stage/nodal status,^21,22^ a biomarker-enriched population with a high relapse rate would be an ideal population for future studies testing new approaches to improve outcomes. Our study suggests that early post-treatment timepoints could be used to ensure timely trial enrollment and initiation of therapy. For example, patients with detectable HPV ctDNA at the end of CRT could be randomly assigned to standard-of-care versus intensified systemic treatment starting within 4-6 weeks of CRT completion. Alternatively, patients with detectable HPV ctDNA who, on restaging, are found to have localized nodal or distant oligometastases could be treated with ablative local therapy. Consistent with previous evidence in other cancer types,^16^ integrating results from multiple longitudinal timepoints (ie, surveillance approach) resulted in higher sensitivity but at the expense of lower specificity, which could be problematic when selecting patients for treatment escalation.

Only a limited number of studies have investigated serial HPV ctDNA in cervical cancer (Data Supplement). Before our study, the largest study had evaluated 40 patients with cervical cancer, of whom 12 relapsed after definitive CRT.^5^ In that study, dPCR-based HPV16/18 ctDNA detection from serum samples collected at the end of treatment was significantly associated with PFS on multivariable landmark analysis (HR, 14.3).^5^ Of note, there was significant heterogeneity in the timing of sample collection (28 days before to 120 days after the last date of treatment and 120 days before relapse), and only 63% (59 of 94) of patients with HPV-16 or HPV-18 cervical cancer had detectable HPV ctDNA from their baseline serum sample.^5^ During longitudinal follow-up, HPV ctDNA detection provided an average of 10 months (range, 2-15 months) lead time before clinical relapse.^5^ Among 13 relapses in the study by Widschwendter et al,^10^ the lead time was 2.4 months. It remains to be seen what constitutes a clinically meaningful lead time that translates into improved survival and/or salvage rates in cervical cancer and how HPV ctDNA compares with other circulating assays such as squamous cell carcinoma antigen.

In contrast to these previous studies, all patients in the present study were treated with 3D image–guided adaptive brachytherapy. Consistent with results from other prospective studies using magnetic resonance imaging (MRI)–guided adaptive brachytherapy,^1^ we observed a low local failure rate. MRI-guided brachytherapy has changed the treatment paradigm for patients with locally advanced cervical cancer; the majority of relapses are now nodal/distant as opposed to local.^1^ The predominance of distant relapse highlights the potential of HPV ctDNA in selecting patients for intensified systemic treatment.

Strengths of our study include the prospective validation design in patients treated at different sites using modern radiotherapy techniques and serial analysis of HPV ctDNA within Streck plasma using two published state-of-the-art assays. Previous studies using quantitative polymerase chain reaction and either serum or EDTA plasma have reported a much lower pretreatment sensitivity (approximately 21%).^23^ An important driver of HPV ctDNA sensitivity in cervical cancer is the inclusion of high-risk HPV genotypes beyond HPV-16 and HPV-18; in our study, 24% of cases harbored one of nine alternative HPV genotypes that could only have been robustly detected from plasma cell-free DNA using a broad sequencing-based approach. To our knowledge, this is the largest prospective cervical cancer cohort with serial HPV ctDNA analysis. dPCR should become more widely available in lower-/middle-income countries where cervical cancer is most prevalent on the basis of guidance from the WHO.^24^

Limitations include missing timepoints mainly because of study blood collection being on hold during the peaks of the COVID-19 pandemic; no routine clinical HPV testing; and lack of study 3-month FDG-PET scan, the most sensitive imaging study currently available for nodal and distant metastases, for all patients.

In conclusion, HPV-seq enables sensitive HPV detection and genotyping directly from plasma. Persistent HPV ctDNA after CRT is independently associated with inferior PFS in this prospective multicenter validation study. HPV ctDNA testing can be used to identify, as early as at the end of CRT, patients at high risk of recurrence in future treatment intensification trials.

## Data Availability

A data sharing statement provided by the authors is available with this article at DOI https://doi.org/10.1200/JCO.23.00954.
